# The complete chloroplast genome of *Heracleum hemsleyanum* Diels (Apioideae), a traditional medicinal herb in China

**DOI:** 10.1080/23802359.2025.2449718

**Published:** 2025-01-16

**Authors:** Xingwu Luo, Zhang Qiaohui, Zhexian Zhang, Li Lin, Qin Zhanghui, Zhu Zhenxing, Wei Fu

**Affiliations:** a Hubei Key Laboratory of Biological Resources Protection and Utilization, Hubei Minzu University, Enshi, Hubei, China; b Enshi Tujia & Miao Autonomous Prefecture Academy of Agricultural Sciences, Enshi, Hubei, China

**Keywords:** *Heracleum hemsleyanum*, complete chloroplast genome, phylogenetic analysis

## Abstract

Heracleum hemsleyanum Diels is a traditional medicinal herb in China. We reported its first complete chloroplast genome. The chloroplast genome was 146,775 bp in length with 37.53% GC content, containing a large single copy region (LSC, 93,309 bp), a small single copy region (SSC, 17,502 bp), and a pair of inverted repeat regions (IRs, 17,982 bp). Moreover, the chloroplast genome encoded 130 genes, including 86 protein-coding genes (PCGs), 36 transfer RNA genes (tRNAs), and eight ribosomal RNA genes (rRNAs). Phylogenetic analysis indicated that H. hemsleyanum was closely related to Heracleum moellendorffii and Heracleum yungningense. This assembled chloroplast genome will provide vital information on the genetic resources, phylogenetic relationships, and the species identification of the genus *Heracleum*.

## 1. Introduction

*Heracleum* L., which is known as hogweed, belongs to the Apiaceae family and is a widespread, taxonomically* complex genus, including approximately 70 ∼ 90 species of biennial or perennial herbs (She et al. 2005; Porrello et al. 2024). Numerous studies reported that several *Heracleum* species have been commonly used in traditional Chinese medicine, spices, and food additives (Gürbüz et al. 2019). For example, *Heracleum moellendorffii* has been used as wild vegetables, and its roots have traditionally been used to treat diseases related to chronic inflammation (Alam et al. 2016; Kim et al. 2019). *Heracleum persicum* has been used as food additives, food preservatives, and flavoring agents (Bahadori et al. 2016; Majidi et al. 2018), and species for the treatment purposes of gastrointestinal, neurological, respiratory, urinary, and rheumatologically dysfunctions (Hajhashemi et al. 2009; Alkan and Celik 2018).

*Heracleum hemsleyanum* Diels 1900, a flowering plant of the genus *Heracleum* (Apioideae), naturally grows under shady forests or moist thickets at an altitude of 2,000 ∼ 3,000 m with cold resistance and is mainly distributed in Southwestern China, such as Hubei, Sichuan, and Yunnan Provinces (She et al. 2005). As a traditional medicinal herb, the roots of *H. hemsleyanum* have a variety of medicinal functions, including treating numbness in the waist and knees, limb cramps, and vitiligo (Wu 1988; Fang et al. 2004). Current research on *H. hemsleyanum* has only focused on its chemical composition and biological functions (Rao et al. 1994; Fang et al. 2004). However, there is limited molecular data in the GenBank for the taxonomy research on *H. hemsleyanum*. Chloroplast is inherited maternally in angiosperm plant species, whose genome can provide abundant informative molecular evidence to resolve taxonomic issues (Niu et al. 2018; Li et al. 2020). Therefore, we reported the first complete chloroplast genome of *H. hemsleyanum* to provide vital information on the genetic resources, phylogenetic relationships, and the species identification of the genus *Heracleum*.

## 2. Materials

**Figure 1.**
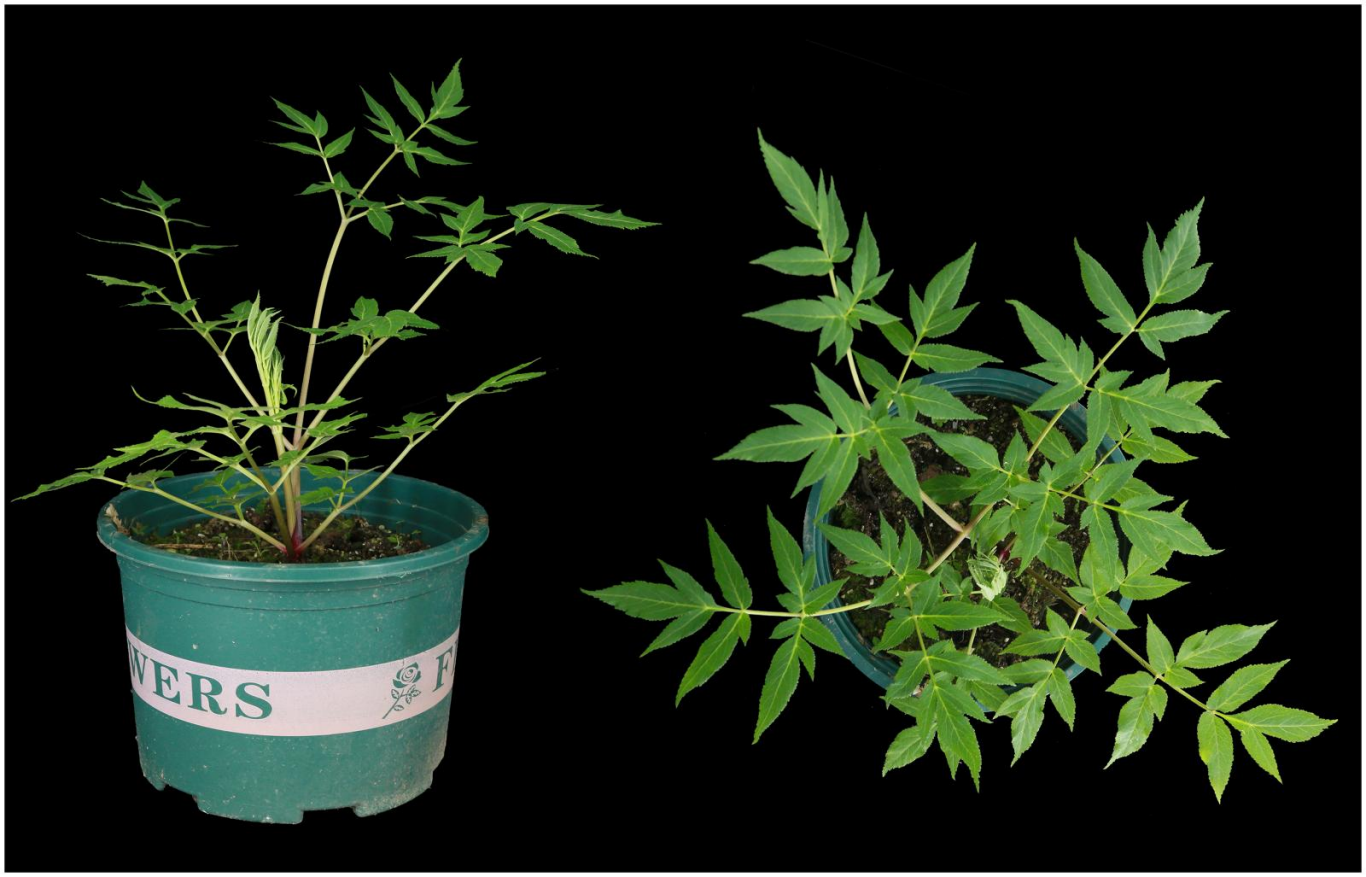
Species reference image of *H. hemsleyanum*. (species images were taken by Qiaohui Zhang and Lin Li at Banqiao, Enshi, Hubei Province, China, 2024).

Samples of *H. hemsleyanum* were collected from Banqiao, Enshi, Hubei Province, China (N 30^o^58’82′’, E 109^o^28’03′’) (Figure 1). A specimen was deposited at the Herbarium of Enshi Tujia & Miao Autonomous Prefecture Academy of Agricultural Sciences (Contact: Wei Fu; fuwei5@mail2.sysu.edu.cn) under the voucher number *DH-20230901*.

## 3. Methods

Total genomic DNA was extracted from the fresh leaves using the modified CTAB method (Doyle and Doyle 1987) and then randomly sheared to yield approximately 350 bp fragments by sonication for library construction. The library was constructed using the primers listed in Supplementary Table 1, and its quality was assessed on the Agilent 5400 system. Paired-end sequencing was performed on the DNBSEQ-T7 platform (Beijing Biomics Tech Co., Ltd, China), yielding approximately 3.24 GB of raw data. After filtering out the low-quality reads and adapter sequences using fastp (Chen et al. 2018), 43,223,208 clean reads were obtained. Then, the complete chloroplast genome of *H. hemsleyanum* was *de novo* assembled using GetOrganelle (version 1.7.1) (Jin et al. 2020). With the *H. millefolium* chloroplast genome (MW228410) (Ciren et al. 2022) as the reference, the assembled genome was annotated using CPGAVAS2 (http://47.96.249.172:16019/analyzer/annotate) (Shi et al. 2019). CPGView (http://47.96.249.172:16085/cpgview/view) (Liu et al. 2023) was used to improve annotation, visualize the structure of the chloroplast genome, and identify gene structure, including *cis*-splicing genes and *trans*-splicing genes. Finally, simple sequence repeat (SSR) loci in the chloroplast genome were identified using MISA (https://webblast.ipk-gatersleben.de/misa/) (Beier et al. 2017) with 10, 6, 5, 5, and 5 as the minimum number of repeats for mono-nucleotide, din-, tri-, tetra-, and penta-, respectively.

To investigate the phylogenetic relationship of *H. hemsleyanum*, eleven complete chloroplast genome sequences were downloaded from the GenBank database. A total of 78 protein-coding genes shared by all genomes were found and extracted using PhyloSuite (version 1.2.2) (Zhang et al. 2020), and then MAFFT (Version 7.407) (Katoh and Standley, 2013) was used for separate alignment of each gene. Then, the aligned sequences were end-to-end concatenated to form a supergene of each species using PhyloSuite (version 1.2.2) (Zhang et al. 2020). With *Panax ginseng* (MH049735) (Wang et al. 2018) as the outgroup, a maximum-likelihood (ML) phylogenetic tree was constructed by IQtree (Version 1.7) (Nguyen et al. 2015) under the TVM+F + I + G4 model.

## 4. Results

**Figure 2.**
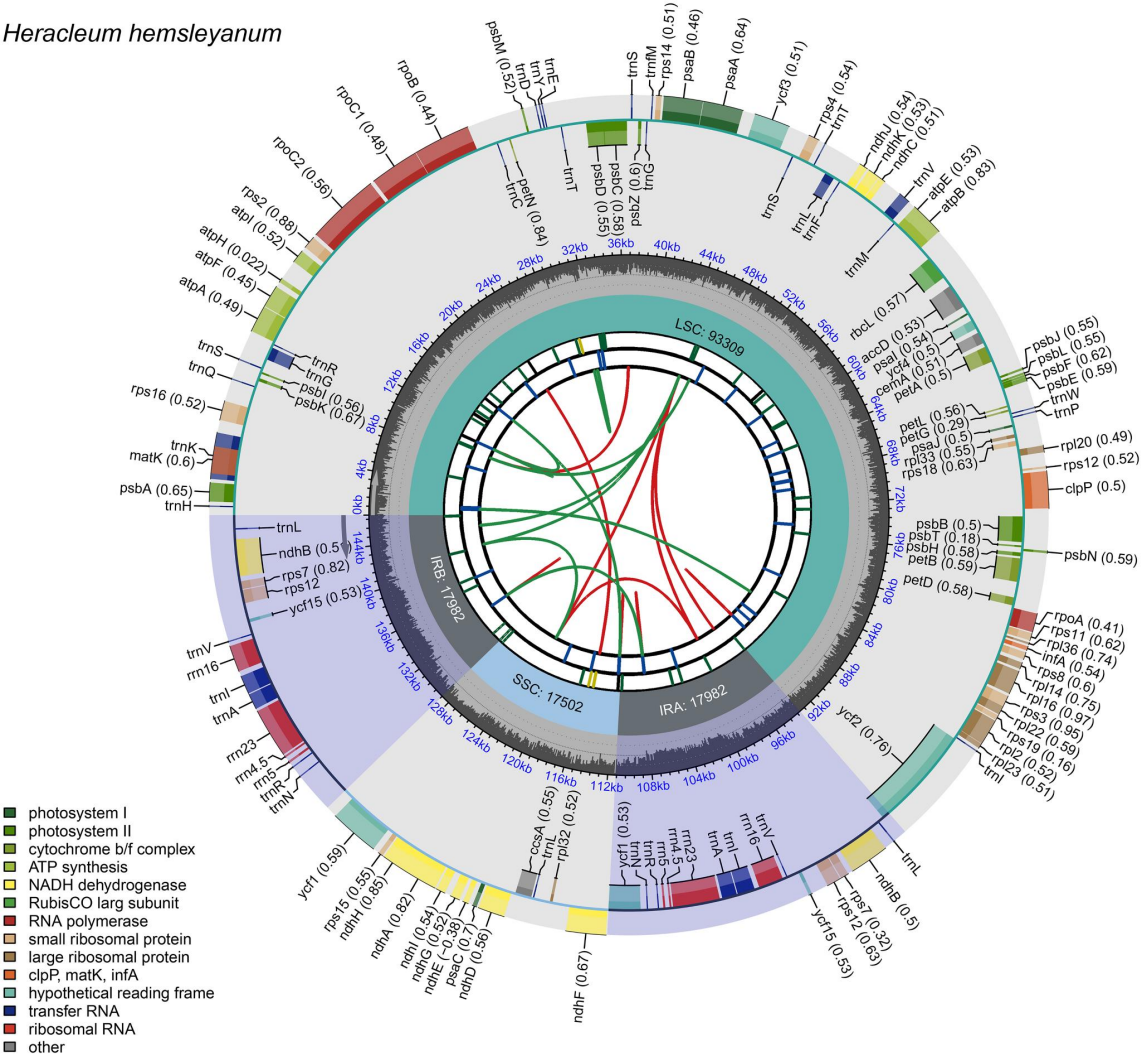
Genetic map of the chloroplast genome of *H.hemsleyanum*. The map consists of six tracks. From the center to the outer, the first track shows dispersed repeats connected by red and green arcs indicating the direction (forward and reverse, respectively). The second track shows long tandem repeats as blue bands, and the third track shows short tandem repeats or microsatellites as green bands. The fourth track represents the GC content along the plastome. Finally, the sixth track represents the genes as colored boxes, the inner boxes present clockwise transcription, and the outer boxes present counterclockwise transcribed genes.

The complete chloroplast genome of *H. hemsleyanum* (accession number: OR823204) was a typical quadripartite structure with 146,775 base pairs (bp) in length, which contained a pair of inverted repeat (IR) regions (17,982 bp) separated by a large single copy (LSC) region (93,309 bp) and a small single copy (SSC) region (17,502 bp), respectively (Figure 2). The Guanine-Cytosine (GC) content of the complete chloroplast genome was 37.53%, while the corresponding values in the LSC, SSC, and IR regions were 35.94%, 31.01%, and 44.83%, respectively. The average coverage depth of the *H. hemsleyanum* chloroplast genome is shown in Supplementary Figure 1. A total of 130 genes were annotated across the entire chloroplast genome, including 86 protein-coding genes (PCGs), 36 transfer RNA genes (tRNAs), and eight ribosomal RNA genes (rRNAs).There were 19 genes containing a single intron, including nine PCG genes (*atpF*, *ndhA*, *ndhB* (x2), *petB*, *rpl16*, *rpl2*, *rpoC1*, and *rps16*) and eight tRNA genes (*trnA-UGC* (x2), *trnG-UCC*, *trnI-GAU* (x2), *trnK-UUU*, *trnL-UAA*, and *trnV-UAC*), while two PCG genes (*ycf3* and *clpP*) had two introns. Eleven *cis*-splicing genes (*rps16*, *atpF*, *rpoC1*, *ycf3*, *clpP*, *petB*, *rpl16*, *rpl2*, *ndhB* (x2), and *ndhA*) and the *trans*-splicing gene (*rps12*) were identified in this chloroplast genome, and their structures are shown in Supplementary Figure 2. In this study, a total of 54 SSRs were identified in the chloroplast genome *H. hemsleyanum*, including 43 mononucleotides and 11 dinucleotides (Supplementary Table 2). Among them, most of the SSRs (37, 86.05%) were composed of A/T base components (Supplementary Table 3). Moreover, over half of the SSRs (32) were located in the LSC region, whereas low proportions of SSRs were found in the SSC or IR regions (Supplementary Table 2).

**Figure 3.**
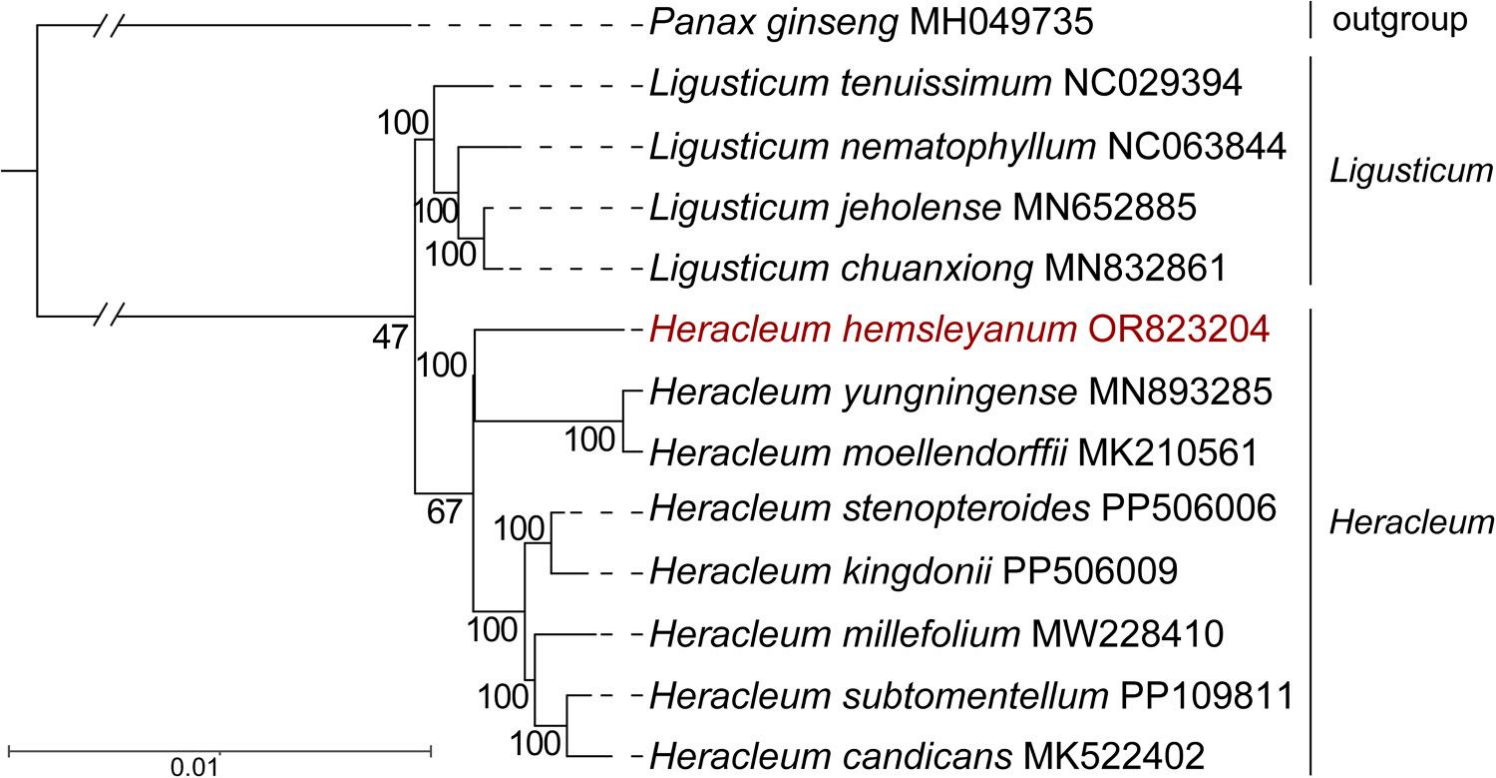
Phylogenetic relationships of *H. hemsleyanum* based on the maximum-likelihood (ML) analysis of 78 protein-coding genes in chloroplast genomes. Bootstrap values next to the nodes are based on 1000 replications. The following sequences were used: *Panax ginseng* MH049735 (Wang et al. 2018), *Ligusticum tenuissimum* NC 029394, *Ligusticum nematophyllum* NC 063844, *Ligusticum chuanxiong* MN832861 (Yuan et al. 2021), *Ligusticum jeholense* MN652885, *Heracleum yungningense* MN893285 (Zheng et al., 2020), *Heracleum moellendorffii* (MK210561), *Heracleum stenopteroides* PP506006, *Heracleum kingdonii* PP506009, *Heracleum millefolium* MW228410 (Ciren et al. 2022), *Heracleum subtomentellum* PP109811 and *Heracleum candicans* MK522402 (Kang et al. 2019).

To further study the phylogenetic position of *H. hemsleyanum*, an ML phylogenetic tree was constructed by IQtree (Version 1.7) (Nguyen et al. 2015). As shown in Figure 3, all the *Ligusticum* species and *Heracleum* species in our study formed a monophyletic group diverging from the outgroup, and *Heracleum* species were further divided into two clades with high bootstrap values. Namely, *H. hemsleyanum* (OR823204), *H. yungningense* (MN893285), and *H. moellendorffii* (MK210561) clustered in one clade, and other *Heracleum* species clustered into another clade. Additionally, *H. hemsleyanum* was closely related to *H. moellendorffii* and *H. yungningense*, which belong to the subgenus *Heracleum*. These results based on the chloroplast genome reported here would facilitate phylogenetic and population genetic diversity of *H. hemsleyanum*.

## 5. Discussion and conclusions

In the current study, the complete chloroplast genome sequence of *H. hemsleyanum* was first sequenced and found to exhibit a typical quadripartite structure with 146,775 bp in length, including an LSC region (93,309 bp), an SSC region (17,502 bp), and a pair of IR regions (17,982 bp). In total, 130 genes were annotated, including 86 PCG genes, 36 tRNA genes, and eight rRNA genes. These features are not significantly different from those of most chloroplast genomes in *Heracleum* (Xiao et al. 2019; Zheng et al. 2020; Ciren et al. 2022). Phylogenetic analysis showed that all the *Heracleum* species in our study were divided into two clades with high bootstrap values. Moreover, *H. hemsleyanum* was closely related to *H. moellendorffii* and *H. yungningense*, which belong to the subgenus *Heracleum*. Our ML analysis results effectively reflect their relationships and are consistent with the traditional taxonomy.

*Heracleum* L. is a large-scale genus belonging to the Apiaceae family and is a widespread, taxonomically complex genus (She et al. 2005; Porrello et al. 2024). Analysis of the chloroplast genome helps understand their relationships and study the phylogeny. The complete chloroplast genome sequence presented in this study would provide basic information on plastome evolution and help infer phylogenetic relationships and the species identification of the genus *Heracleum*. In the future, increasing the number of chloroplast genomes of *Heracleum* species will provide deeper insights into the evolution of this important family.

## Supplementary Material

Supplementary Tables.docx

## Data Availability

The genome sequence data that support the findings of this study are openly available in GenBank of NCBI at (https://www.ncbi.nlm.nih.gov/) under the accession no. OR823204. The associated BioProject, SRA, and Bio-Sample numbers are PRJNA1041483, SRR26857948, and SAMN38286218, respectively.
