# Supplementary material for: The complete chloroplast genome of Heracleum hemsleyanum Diels (Apioideae), a traditional medicinal herb in China
Source: Mitochondrial DNA B Resour. 2025 Jan 16;10(2):114–8. doi: 10.1080/23802359.2025.2449718 (PMC11740293; doi:10.1080/23802359.2025.2449718)
Supplement: Supplementary Tables.docx [file TMDN_A_2449718_SM6812.docx]

**Supplementary Table 1.** The PCR primers were used for DNA library construction.

| **Primer name** | **Sequences** |
| --- | --- |
| p7_1 | GATCGGAAGAGCACACGTCTGAACTCCAGTCACATCACGATCTCGTATGCCGTCTTCTGCTTG |
| p5 | AGATCGGAAGAGCGTCGTGTAGGGAAAGA |

**Supplementary Table 2.** Simple sequence repeats (SSRs) in *Heracleum hemsleyanum* chloroplast genome.

| **Number** | **Type** | | **SSR** | **Size** | **Start** | **End** | **Location** |
| --- | --- | --- | --- | --- | --- | --- | --- |
| 1 | p1 | (A)10 | | 10 | 4655 | 4664 | LSC |
| 2 | p1 | (G)16 | | 16 | 9200 | 9215 | LSC |
| 3 | p1 | (T)10 | | 10 | 9341 | 9350 | LSC |
| 4 | c | (AT)6tatt(TA)18 | | 52 | 10279 | 10330 | LSC |
| 5 | p1 | (T)11 | | 11 | 12827 | 12837 | LSC |
| 6 | c | (A)10gaacagagttctttttgtatcgtcctgccccccctttgatatatttg(AT)8 | | 73 | 13444 | 13516 | LSC |
| 7 | p1 | (A)10 | | 10 | 13987 | 13996 | LSC |
| 8 | p1 | (T)10 | | 10 | 14949 | 14958 | LSC |
| 9 | c | (AT)11ttata(AT)8 | | 43 | 15936 | 15978 | LSC |
| 10 | p1 | (A)15 | | 15 | 16866 | 16880 | LSC |
| 11 | p1 | (T)11 | | 11 | 19089 | 19099 | LSC |
| 12 | p1 | (A)10 | | 10 | 21727 | 21736 | LSC |
| 13 | c | (TA)8(T)11 | | 27 | 23261 | 23287 | LSC |
| 14 | p1 | (T)12 | | 12 | 26837 | 26848 | LSC |
| 15 | p1 | (T)11 | | 11 | 29990 | 30000 | LSC |
| 16 | p2 | (AT)6 | | 12 | 30449 | 30460 | LSC |
| 17 | p1 | (T)13 | | 13 | 33119 | 33131 | LSC |
| 18 | p1 | (T)11 | | 11 | 33467 | 33477 | LSC |
| 19 | p1 | (A)11 | | 11 | 33681 | 33691 | LSC |
| 20 | p1 | (T)10 | | 10 | 45623 | 45632 | LSC |
| 21 | p1 | (A)17 | | 17 | 46005 | 46021 | LSC |
| 22 | p1 | (C)10 | | 10 | 52590 | 52599 | LSC |
| 23 | c | (T)10caagcgtggaagcccccgaactagaattagtaggatttattttcataataataataaataaagtcaaataggtcgaaa(T)10 | | 98 | 56031 | 56128 | LSC |
| 24 | p1 | (A)13 | | 13 | 56617 | 56629 | LSC |
| 25 | p1 | (T)10 | | 10 | 58399 | 58408 | LSC |
| 26 | p1 | (T)10 | | 10 | 60950 | 60959 | LSC |
| 27 | p1 | (T)13 | | 13 | 61176 | 61188 | LSC |
| 28 | p1 | (A)12 | | 12 | 64519 | 64530 | LSC |
| 29 | p1 | (T)12 | | 12 | 71521 | 71532 | LSC |
| 30 | p1 | (T)13 | | 13 | 73086 | 73098 | LSC |
| 31 | p1 | (A)11 | | 11 | 76394 | 76404 | LSC |
| 32 | p1 | (T)16 | | 16 | 85278 | 85293 | LSC |
| 33 | p1 | (G)10 | | 10 | 93934 | 93943 | IRa |
| 34 | p1 | (T)15 | | 15 | 98985 | 98999 | IRa |
| 35 | p1 | (T)10 | | 10 | 102971 | 102980 | IRa |
| 36 | p1 | (G)10 | | 10 | 104212 | 104221 | IRa |
| 37 | p1 | (A)10 | | 10 | 110808 | 110817 | IRa |
| 38 | c | (AT)6aaaatatataa(AT)6 | | 35 | 111303 | 111337 | SSC |
| 39 | p2 | (TA)8 | | 16 | 114486 | 114501 | SSC |
| 40 | p2 | (AT)9 | | 18 | 115121 | 115138 | SSC |
| 41 | p1 | (A)13 | | 13 | 116211 | 116223 | SSC |
| 42 | p1 | (T)14 | | 14 | 127597 | 127610 | SSC |
| 43 | p1 | (T)11 | | 11 | 128077 | 128087 | SSC |
| 44 | p1 | (T)10 | | 10 | 129268 | 129277 | IRb |
| 45 | p1 | (C)10 | | 10 | 135864 | 135873 | IRb |
| 46 | p1 | (A)10 | | 10 | 137105 | 137114 | IRb |
| 47 | p1 | (A)15 | | 15 | 141086 | 141100 | IRb |
| 48 | p1 | (C)10 | | 10 | 146142 | 146151 | IRb |

Note: p1, p2, and c represent mononucleotide, dinucleotide, and compound SSR, respectively.

**Supplementary Table 3.** Frequency of complementary simple sequence repeats in *Heracleum hemsleyanum* chloroplast genome.

| **Repeats** | **5** | **6** | **7** | **8** | **9** | **10** | **11** | **12** | **13** | **14** | **15** | **16** | **17** | **18** | **Total** |
| --- | --- | --- | --- | --- | --- | --- | --- | --- | --- | --- | --- | --- | --- | --- | --- |
| A/T | - | - | - | - | - | 15 | 8 | 3 | 5 | 1 | 3 | 1 | 1 |  | 37 |
| C/G | - | - | - | - | - | 5 |  |  |  |  |  | 1 |  |  | 6 |
| AT/AT | - | 4 |  | 4 | 1 |  | 1 |  |  |  |  |  |  | 1 | 11 |
